# Colchicine as a novel drug for the treatment of osteosarcoma through drug repositioning based on an FDA drug library

**DOI:** 10.3389/fonc.2022.893951

**Published:** 2022-08-18

**Authors:** Jisun Oh, Hyun−Ju An, Hyun Jeong Yeo, Sujin Choi, Jisu Oh, Segi Kim, Jin Man Kim, Junwon Choi, Soonchul Lee

**Affiliations:** ^1^ Department of Orthopaedic Surgery, CHA Bundang Medical Center, CHA University School of Medicine, Seongnam-si, South Korea; ^2^ Division of Hemato-Oncology, Department of Internal Medicine, Yongin Severance Hospital, Yonsei University College of Medicine, Yongin-si, South Korea; ^3^ Department of Oral Microbiology and Immunology School of Dentistry, Seoul National University, Seoul, South Korea; ^4^ Department of Molecular Science and Technology, Ajou University, Suwon-si, South Korea

**Keywords:** Saos-2, U2OS, drug repositioning, FDA-approved drugs, colchicine

## Abstract

**Background:**

Colchicine is a traditional medication that is currently approved to treat gout and familial Mediterranean fever (FMF). However, colchicine has a wide range of anti-inflammatory activities, and several studies have indicated that it may be useful in a variety of other conditions, such as rheumatic disease, cardiac disease, and cancer. Osteosarcoma, the most common type of bone sarcoma, is derived from primitive bone-forming mesenchymal cells. In this study, we investigated whether colchicine could be used to treat osteosarcoma through the regulation of cell cycle signaling.

**Methods:**

Two human osteosarcoma cell lines, U2OS and Saos-2, were used. A clonogenic assay was used to determine the antiproliferative effects of colchicine on osteosarcoma cells. Reactive oxygen species (ROS) production and apoptosis were measured by flow cytometry. Migration and invasion assays were performed to investigate the inhibitory effects of colchicine. The signaling pathways related to colchicine treatment were verified by GO biological process (GOBP) and Kyoto Encyclopedia of Genes and Genomes (KEGG) enrichment analyses.

**Results:**

Colchicine was selected as the lead compound based on the results of initial screening and cell viability assays conducted in Saos-2 and U2Os cells. Colchicine reduced the viability of Saos-2 and U2OS cells in a concentration-dependent manner. It also significantly inhibited colony-forming ability and induced ROS production and apoptosis. It also inhibited the migration and invasion of both Saos-2 and U2OS cells. GOBP and KEGG enrichment analyses indicated the involvement of microtubule-based processes and cancer-related pathways.

**Conclusions:**

These findings suggest that colchicine has therapeutic potential in osteosarcoma.

## Introduction

Osteosarcoma is a relatively rare bone tumor with a global incidence of 3.4 cases per million people per year (1). It is typically diagnosed at a young age, with a second peak in incidence seen in elderly individuals (2). While surgical techniques and adjuvant chemotherapies have ameliorated poor outcomes for deadly cancers, the 5-year survival rate of osteosarcoma has plateaued over the past four decades (3). Current chemotherapies used to treat osteosarcoma include methotrexate, doxorubicin, cisplatin, carboplatin, ifosfamide, cyclophosphamide, etoposide, and gemcitabine. Usually, two or more drugs are administered in combination, and many experts recommend that the drugs be administered at very high doses when possible (4). Limiting factors associated with current treatments include complications, fatal toxicity, and resistance, which highlights the need to develop novel therapeutics for osteosarcoma.

Drug repositioning is an efficient tool to identify new uses for existing drugs in a cost-effective and time-saving manner (5). Drug repositioning has recently emerged as a potential source of new anticancer treatments. The heterogenic nature of osteosarcoma can lead to the failure of anticancer treatment, suggesting that approved drugs may be used to interfere with diverse targets within cancer cells more effectively (6). Drug repositioning has found that thalidomide, a sedative hypnotic agent, is effective against multiple myeloma (7). In addition, nonsteroidal anti-inflammatory drugs (NSAIDs) with antiplatelet effects have been demonstrated to be effective against colorectal cancer (8). Metformin, a drug for type 2 diabetes, is effective against endometrial cancer, and digoxin, a cardiac glycoside, has efficacy against prostate cancer (9, 10). Nonetheless, the development of novel therapeutics for osteosarcoma through drug repositioning remains to be explored. Thus, discovering a new pharmacological effect for a drug in which human safety is already established and expanding its therapeutic range to osteosarcoma would be beneficial for developing new anticancer treatments for osteosarcoma.

Colchicine is a first-line therapy for treatment of acute gout, prophylaxis of gout, and familial Mediterranean fever (FMF) (11). According to previous studies, colchicine inhibits microtubule polymerization and stimulates cells to enter mitosis and arrest with condensed chromosomes (12). Studies have uncovered the benefits of colchicine and determined the underlying mechanisms by which colchicine acts in a growing variety of diseases. Colchicine has recently been demonstrated to play a role in cardiac diseases such as pericarditis and coronary artery disease (13), and it is also routinely used in other rheumatic diseases, including for acute flares in calcium pyrophosphate deposition disease (CPPD) (14). Thus, colchicine may be effective in treating various diseases. Therefore, we investigated the effects of colchicine on osteosarcoma cell lines in the context of its contribution to cell cycle signaling.

## Materials and methods

### Reagents and antibodies

SCREEN-WELL (FDA-approved drug library V2) was purchased from Enzo Life Sciences (Enzo, NY, USA). Dulbecco’s modified Eagle’s medium (DMEM) and Roswell Park Memorial Institute (RPMI) 1640 medium were purchased from Gibco BRL (Grand Island, NY). Fetal bovine serum (FBS) was purchased from GenDEPOT (Barker, TX). Primary antibodies for cleaved caspase 3 and cleaved PARP were purchased from Cell Signaling Technology (Danvers, MA). Antibodies against β-actin were purchased from Santa Cruz Biotechnology (Dallas, TX). Bicinchoninic acid (BCA) protein assay reagent was purchased from Pierce Biotechnology (Rockford, IL).

### Cell culture

Human osteosarcoma Saos-2 and U2OS cell lines, obtained from the Korean Cell Line Bank (Seoul, Korea), were maintained at 37°C in a humid atmosphere of 5% CO_2_ and 95% air in RPMI and DMEM, respectively. Each medium contained 10% FBS and 1% antibiotics.

### Cologenic assay

Saos-2 and U2OS cells were plated in six-well plates at densities of 500 and 300 cells per well, respectively. After 24 h, they were treated with different concentrations of colchicine for 48 h. The medium was then changed, and the cells were incubated for an additional 12 days in drug-free medium. The colonies that formed were fixed with 3.7% formaldehyde at room temperature for 10 min and then stained with 0.05% crystal violet. Colonies with >10 cells were counted under a light microscope. The experiments were carried out in triplicate.

### Intracellular reactive oxygen species

Intracellular ROS levels were measured by staining the cells with 2-7-dichlorofluorescein diacetate (DCF-DA; Molecular Probes). Cells were treated with DMSO or colchicine for 48 h and treated with 5 μM DCF-DA 37°C for 1 h. The fluorescence intensity of the cells was monitored *via* flow cytometry using a FACSCalibur apparatus (BD Biosciences, San Jose, CA).

### Apoptosis analysis

Saos-2 and U2OS cells were collected, washed with phosphate-buffered saline (PBS), and dissociated with Accutase solution (Sigma-Aldrich, St. Louis, MO). The cells were then counted and washed with PBS containing 2% FBS and 0.1% Tween-20. Apoptosis in cultured cells was assessed by flow cytometry after Annexin V-FITC/propidium iodide (PI) double staining using the Annexin-V FITC Apoptosis Detection Kit I (BD Biosciences, San Jose, CA) according to the manufacturer’s instructions.

### Western blot analysis

The cells were washed with cold PBS, and the pellet was collected. Cells were then suspended in 1× cell lysis buffer (Cell Signaling Technology, Danvers, MA) enriched with 0.1 mM PMSF and protease inhibitor (Roche, Basel, Switzerland) and incubated on ice for 30 min. After centrifugation at 13,000 *g* for 15 min, the supernatant was collected as a whole cell lysate. Lysates containing equal amounts of protein were prepared and analyzed using sodium dodecyl sulfate–polyacrylamide gel electrophoresis (SDS-PAGE). The blots were then blocked with 5% fat-free dry milk–TBST (Tris-based saline buffer containing 0.1% Tween-20) for 1 h at room temperature and probed overnight using the indicated primary antibody (1/5,000 dilution for β-actin and 1/1,000 dilution for the rest). The blots were then washed three times with TBST buffer for 10 min each. The washed blots were then incubated with 1:5,000 diluted horseradish peroxidase-conjugated secondary antibodies (Thermo Fisher Scientific, MA, USA). The blots were washed three times with TBST buffer for 10 min each, and the transferred proteins were detected after incubation with the ECL substrate detection reagent.

### Transwell assay

Transwell migration assays were used to determine the migratory and invasive abilities of osteosarcoma cells after colchicine treatment. Saos-2 cells (1 × 10^4^ cells) and U2OS cells (5 × 10^3^ cells) were seeded into the apical side of the transwell chamber with a non-coated membrane using an 8-μm pore (Corning, NY, USA). For invasion assays, Saos-2 and U2OS cells were plated at the same density as mentioned above with a Matrigel-coated membrane (BD Biosciences, San Jose, CA, USA). To create a chemotactic gradient, serum-free media were added to the upper chamber, while 10% FBS was added to the lower chamber media. After 48 h, the cells were fixed in methanol for 5 min, stained with 0.05% crystal violet, washed with PBS, and allowed to dry. Images of the cells that migrated through the pores were taken with an optical microscope, and the cells were counted. Migration and invasion assays were carried out in sextuple and triplicate, respectively.

### The GOBP and KEGG enrichment analyses

Library preparation and sequencing of control or colchicine-treated total RNAs and library construction were performed using the QuantSeq 3’ mRNA-Seq Library Prep Kit (Lexogen, Inc., Austria) according to the manufacturer’s instructions. In brief, 500 ng of total RNA was prepared for each sample, an oligo-dT primer containing an Illumina-compatible sequence at its 5’ end was hybridized to the RNA, and reverse transcription was performed. After degradation of the RNA template, second-strand synthesis was initiated by a random primer containing an Illumina-compatible linker sequence at its 5’ end. The double-stranded library was purified using magnetic beads to remove all reaction components. The library was amplified to add the complete adapter sequences required for cluster generation. The final library was purified from the PCR components. High-throughput sequencing was performed as single-end 75 sequencing using a NextSeq 500 (Illumina, Inc., USA). Data analysis of QuantSeq 3’ mRNA-Seq reads was aligned using Bowtie2 (15). Bowtie2 indices were either generated from the genome assembly sequence or the representative transcript sequences for alignment to the genome and transcriptome. The alignment file was used for assembling transcripts, estimating their abundance, and detecting the differential expression of genes. Differentially expressed genes were determined based on the counts from unique and multiple alignments using coverage in Bedtools (16). The read count (RC) data were processed based on the quantile normalization method using EdgeR within R (R Development Core Team, 2016) using Bioconductor (17). Gene classification was based on searches of the DAVID and Medline databases. Data mining and graphic visualization were performed using ExDEGA (Ebiogen Inc., Korea).

### Cell viability assay

Cell viability was detected using a CCK-8 solution (Dojindo, Kumamoto, Japan) according to the manufacturer’s protocol. Briefly, the Saos-2 and U2OS cells were counted and seeded at a density of 5 × 10^4^ cells/well in 96-well plates. One day after seeding, cells were incubated with different concentrations (0.01, 0.05, 0.1, 0.5, 1, and 5 μM) of colchicine. After 48 h of treatment, 10 μl of CCK-8 was added, and the plates were incubated at 37°C for 2 h. The absorbance per well was measured at a wavelength of 450 nm. Each experiment was sextupled independently.

### Animal study

All animal experiments were conducted in accordance with the Guide for the Care and Use of Laboratory Animals. Five-week-old BALB/c nude mice were acclimatized for 1 week under standard temperature, humidity, and timed lighting conditions at the animal care facility. Five mice were used for each group. Saos-2 cells were injected subcutaneously into the flank at a density of 5 × 10^6^ cells/ml in PBS/Matrigel (7:3, v/v) in a total volume of 100 µl. The mice were divided into control and treatment groups once the tumor volume reached 150 mm^3^. Vehicle (PBS) or colchicine (10 μM) was injected (i.t.) at 100 µl. Tumor volumes were measured every 3–4 days from the day of colchicine injection for 15 days. Tumor volumes were calculated using the following formula: length × width × 0.52. The mice were sacrificed 15 days after colchicine treatment.

### Statistical analysis

Data were analyzed using the GraphPad Prism 6 software and are expressed as mean ± standard deviation. A Mann–Whitney *U* test was used to compare the means of two groups and a Kruskal–Wallis test with Tukey’s *post-hoc* test was used when the means of two or more variables (i.e., induction and concentration) were compared. Results were considered significant when *p* < 0.05.

## Results

### Initial screening of an FDA-approved library identified colchicine as a potential drug for reducing the viability of osteosarcoma cells

**Figure 1:**
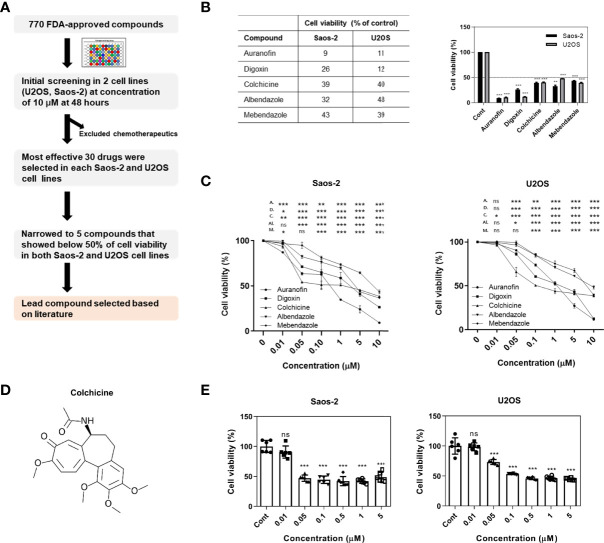
Initial screening of an FDA-approved library and colchicine as a potential drug for osteosarcoma treatment. **(A)** A schematic representation of the workflow implemented to select colchicine. **(B)** The cell viability assessed by CCK-8 assay. Five compounds that showed below 50% of cell viability (% of control) with a concentration of 10 μM in Saos-2 and U2OS cells. The results are presented as means ± SD (*n* = 2). **p < 0.01, ***p < 0.001. **(C)** Saos-2 and U2OS cells were treated with five compounds (0.01, 0.05, 0.1, 0.5, 1, 5, and 10 μM) or vehicle for 48 h. The data are presented as means ± SD (*n* = 3) A., D., C., AI., and M. represent auranofin, digoxin, colchicine, albendazole, and mebendazole, respectively. NS, not significant. *p < 0.05, **p < 0.01, ***p < 0.001. **(D)** Chemical structure of colchicine. **(E)** Saos-2 and U2OS cells were treated with colchicine (0.01, 0.05, 0.1, 0.5, 1, and 5 μM) or vehicle for 48 h. The data are presented as means ± SD (*n* = 6). NS, not significant. ***p < 0.001.

We assessed 772 compounds in an FDA-approved library for their ability to reduce the viability of osteosarcoma cells. Two human osteosarcoma cell lines (U2OS and Saos-2) were used to select the lead compound (**Figure 1A**). Initially, osteosarcoma cell lines were treated with each compound at a concentration of 10 μM for 48 h and screened using the CCK-8 assay. Compounds that were originally developed as chemotherapeutics were excluded. Thirty compounds that demonstrated the most effective inhibitory effects were selected from both Saos-2 and U2OS cell lines for a second screening. Subsequently, five compounds were identified that inhibited the growth of both Saos-2 and U2OS cells to below 50% compared to that of the control (**Figure 1B**), and treated with different concentrations (0.01, 0.05, 0.1, 0.5, 1, 5, and 10 μM) to confirm the dose-dependent cytotoxic effect (**Figure 1C**). Based on previous research and clinical use, colchicine was selected as the lead compound (**Figure 1D**). Colchicine has been actively investigated beyond the scope of its original use, including in cancer (18–21). In line with these previous studies, colchicine was of prime interest among all the candidates tested. We examined the effects of colchicine on the viability of Saos-2 and U2OS cells. Treatment of the cells with colchicine for 48 h resulted in cytotoxic effects in both osteosarcoma cell lines (**Figure 1E**).

### Colchicine inhibits proliferation and induces ROS production and apoptosis in osteosarcoma cells

**Figure 2:**
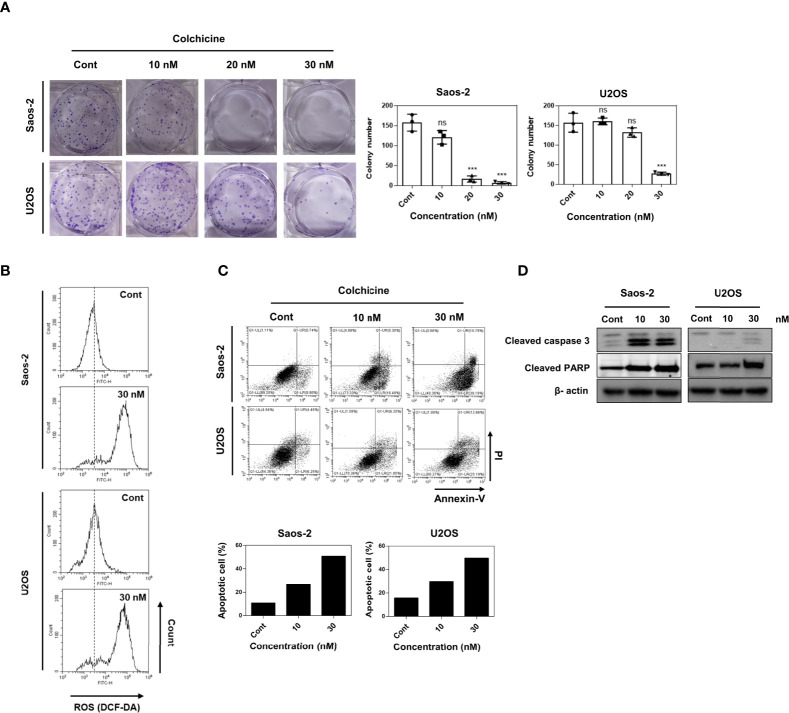
Effects of colchicine on colony-forming ability, ROS production, and apoptosis in Saos-2 and U2OS cells. **(A)** Saos-2 and U2OS cells were treated with three different concentrations of colchicine (10, 20, or 30 nM) or vehicle (DMSO) and were seeded in a six-well plate. The colony size >100 μm was counted under a light microscope. Results are presented as means ± SD (*n* = 3). NS, not significant, ***p < 0.001. **(B)** Measurement of ROS levels through flow cytometry with 2’7’-dichlorodihydrofluorescein diacetate assay after treatment with colchicine (30 nM) or vehicle (DMSO) for 48 h on Saos-2 and U2OS cells. **(C)** Flow cytometric analyses of Saos-2 and U2OS treated with different concentrations of colchicine (15 or 30 nM) or vehicle (DMSO) for 48 h carried out after staining with Annexin V-FITC/PI. Data shown are representative images and dot plots are shown for the analyzed cells. **(D)** The expression of cleaved caspase 3 and cleaved PARP were determined after treatment with colchicine (10 or 30 nM) for 48 h in both Saos-2 and U2OS cells. The whole lysates were subjected to Western blot analysis.

Colony-forming assays characterize the ability of cancer cells to grow into colonies through independent growth and clonal expansion. Thus, we evaluated the effect of colchicine on the clonogenic survival of osteosarcoma cells. As shown in **Figure 2A**, different concentrations (20 and 30 nM) and 30 nM of colchicine significantly reduced the colony formation of both Saos-2 and U2OS cells in a dose-dependent manner, respectively. ROS has been suggested to induce cancer cell cycle arrest and apoptosis (22). The effect of colchicine on ROS production in colon cancer (HT-29) cells has already been discussed (19). Therefore, we also hypothesized that we would see a surge in ROS levels in osteosarcoma cells after colchicine treatment. In the 2’,7’-dichlorofluorescin diacetate (DCF-DA) ROS assay, colchicine treatment (30 nM) induced a change in intracellular ROS levels compared to the control (**Figure 2B**) in both Saos-2 and U2OS cells. To examine whether apoptosis impairs cell viability *via* colchicine treatment, flow cytometry analysis with annexin V-FITC/PI was used to assess the percentage of apoptotic cells. Similar to the cell viability assay findings, colchicine significantly reduced the percentage of live cells and increased total apoptosis in both Saos-2 and U2OS cells at 10 and 30 nM (**Figure 2C**). Similarly, treatment with colchicine increased the expression of cleaved caspase 3 and cleaved PARP in Saos-2 and U2OS cells (**Figure 2D**). These results indicated that colchicine exerts anticancer effects by inhibiting proliferation and inducing apoptosis in osteosarcoma cells.

### Colchicine inhibits migration and invasion of osteosarcoma cells

**Figure 3:**
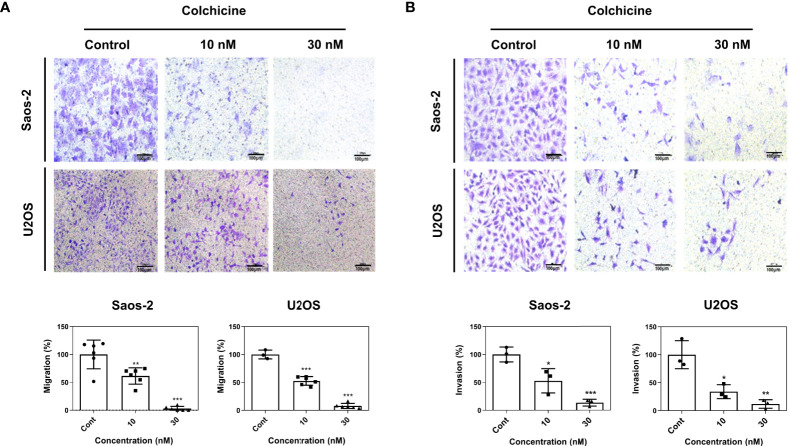
Anti-migration and anti-invasion effects of colchicine in Saos-2 and U2OS cells. **(A)** Representative images of a transwell migration assay and **(B)** a Matrigel-coated transwell invasion assay of Saos-2 and U2OS cells. Both cells were treated with different concentrations of colchicine (10 or 30 nM) or vehicle (DMSO) for 48 h and reseeded on the transwell insert for 48 h before fixation and crystal violet staining. Images of migrated cells were taken under an optical microscope. The values are presented as means ± SD (*n* = 6 and *n* = 3, respectively). *p < 0.05, **p < 0.01, ***p < 0.001.

Enhanced cell mobility and invasiveness are hallmarks of cancer (23). Cell migration and invasion are propelling factors that provide a wide array of cellular mechanisms under the metastatic cascade (24). Therefore, to investigate the role of colchicine in cell migration and invasion, we performed Transwell cell migration and Matrigel invasion assays. Colchicine treatment (10 or 30 nM) significantly inhibited cell migration, as determined by transwell migration of both Saos-2 and U2OS cells (**Figure 3A**). We also found that colchicine treatment (10 or 30 nM) dramatically inhibited the invasion of Saos-2 and U2OS cells (**Figure 3B**). Overall, these results indicate that colchicine inhibits the migration and invasion of osteosarcoma cells *in vitro*.

### Significantly enriched pathways included microtubule-based process and cancer related pathways

**Figure 4:**
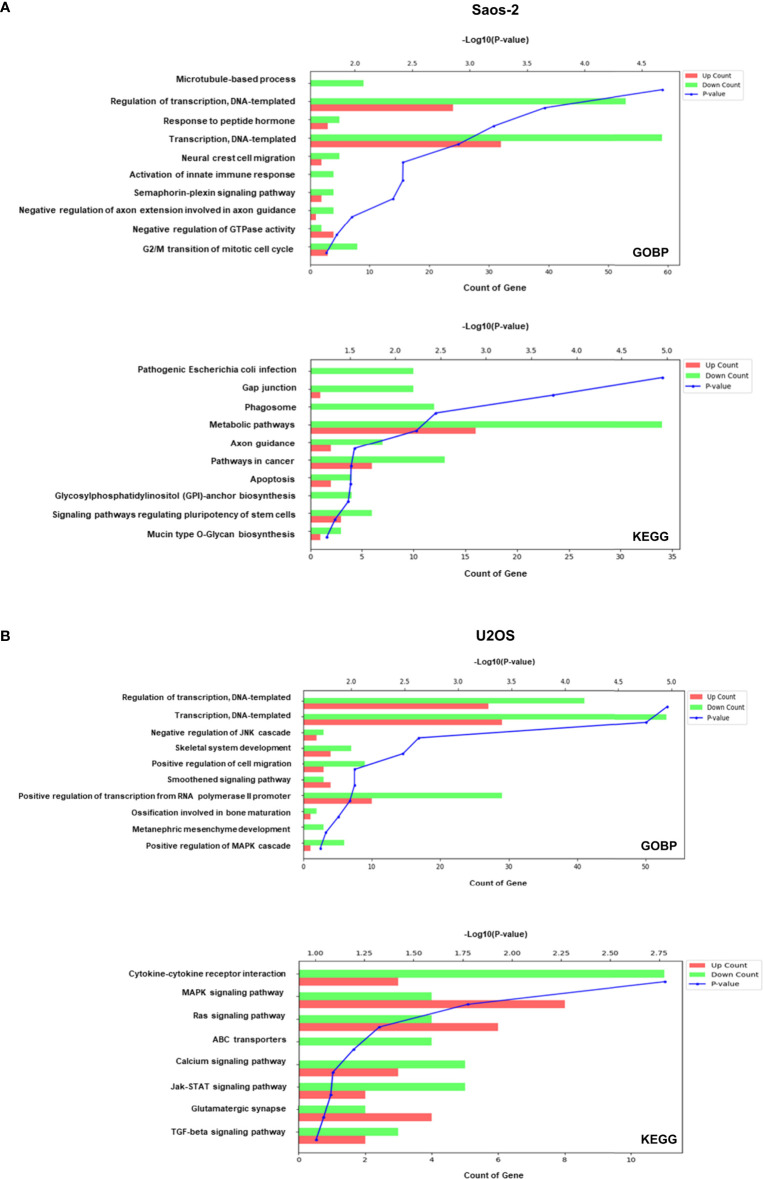
GO biological process (GOBP) and Kyoto Encyclopedia of Genes and Genomes (KEGG) enrichment of genes modulated in Saos-2 and U2OS cells upon colchicine treatment. GOBP (top) and KEGG (bottom) enrichment analysis on upregulated and downregulated proteins of **(A)** Saos-2 cells and **(B)** U2OS cells. The vertical axis represents the pathway category, and the horizontal axis represents the enrichment score [−log(*p*-value)] of the pathway. Significantly enriched GOBP and KEGG pathways (*p* < 0.05) are presented. The data were analyzed using DAVID bioinformatics tools.

To explore relevant biological functions, GO biological process (GOBP) and Kyoto Encyclopedia of Genes and Genomes (KEGG) enrichment analyses were performed on the upregulated and downregulated genes after colchicine treatment and analyzed using the DAVID bioinformatics tools. As shown in **Figure 4A**, there was a significant involvement of gene related to the cell cycle, including microtubule-based process and G2/M transition of the mitotic cell cycle in Saos-2 cells. This finding provides a plausible explanation for the anticancer effects of colchicine through the regulation of microtubule-based processes in osteosarcoma cells. KEGG analysis revealed that the pathways involved in the colchicine-treated cells were related to gap junctions and apoptosis. These data further support the possibility that colchicine induces apoptosis and inhibits the migration and invasion of osteosarcoma cells, as previously described. The GOBP and KEGG analyses in U2OS cells found that colchicine treatment significantly affected cancer-related pathways compared to that of the control (**Figure 4B**).

### Colchicine inhibits osteosarcoma growth *in vivo*

**Figure 5:**
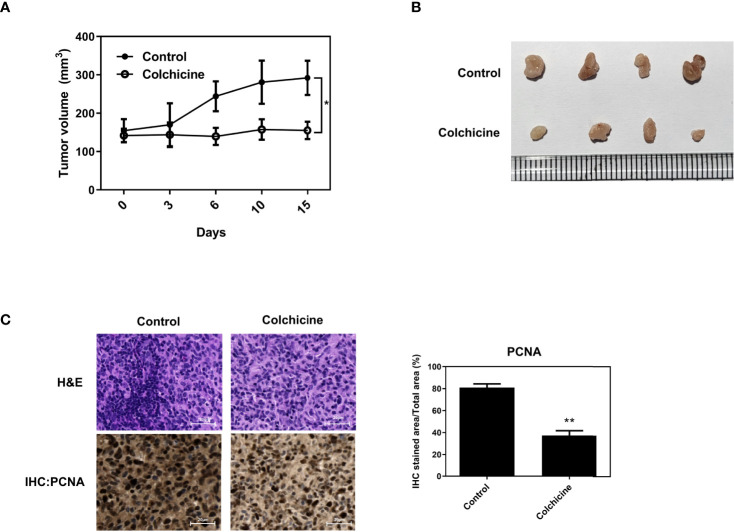
Inhibitory effects of colchicine on Saos-2 growth *in vivo*. Saos-2 cells were injected subcutaneously into the flank of BALB/c nude mice. After tumor injection, colchicine (10 μM) was injected (i.t.) once. **(A)** The tumor volume was measured every 3–4 days from the day of colchicine injection for 15 days. Tumor volume was measured with digital calipers and calculated using the formula 0.52 × length × width. The values are presented as means ± SD (*n* = 5). *p < 0.05. **(B)** The representative photograph of saos-2 xenografts with an intratumoral dose of colchicine (10 μM). Mice were sacrificed after 15 days of the colchicine treatment. **(C)** The effect of colchicine treatment on the expression of proliferating cell nuclear antigen (PCNA) was examined by immunohistochemical analysis. The values are presented as means ± SD (*n* = 3). **p < 0.01.

To verify the inhibitory effect of colchicine on osteosarcoma cells *in vivo*, BALB/c nude mice were injected subcutaneously with Saos-2 cells into the flank. Intraperitoneal administration of colchicine (10 μM) significantly reduced tumor volume compared to that in the vehicle-treated group (**Figures 5A, B**). The inhibitory effect of colchicine on the growth of Saos-2 cells was accompanied by a marked decrease in proliferating cell nuclear antigen (PCNA) expression (**Figure 5C**).

## Discussion

In recent decades, a considerable amount of research has focused on developing new chemotherapeutics. Despite efforts in drug discovery, the development of novel molecules for clinical use has not significantly increased. Thus, recently, there has been an increase in the repositioning of drugs in oncology. Drug repositioning is particularly advantageous in rare tumors, such as osteosarcoma. Based on the initial screening and cytotoxic effects of the FDA-approved compounds, five compounds (auranofin, digoxin, colchicine, albendazole, and mebendazole) were selected as candidates. In this process, compounds that are originally developed as chemotherapeutics for other cancer types were excluded. Under the presumption that chemotherapeutics would naturally exhibit anticancer effects in most of the cancer types including osteosarcoma, they were eliminated for identifying the novel cytotoxic effects of non-chemotherapeutic agents. Auranofin is an oral agent used for the treatment of rheumatoid arthritis. Several studies have reported its anticancer effects in various types of cancers, including osteosarcoma (25–27). Digoxin, an antiarrhythmic agent, has been reported to enhance anticancer effects in non-small cell lung cancer (28). Notwithstanding preclinical evidence demonstrating the anticancer effects of cardiac glycosides, epidemiologic studies have shown a potential risk of breast cancer in digoxin users (29). In agreement with these findings, Chung et al. demonstrated that patients taking digoxin were more likely to develop cancers, including breast, liver, and lung cancers, during a 10-year follow-up period (30). Based on the advanced studies on osteosarcoma and the increased risk of cancer respectively, auranofin and digoxin were excluded as the lead compounds in this study. It has been well demonstrated that colchicine binds to soluble tubulin to form tubulin–colchicine complexes and block mitotic cells in metaphase (31). In this manner, cancer cells are more susceptible to colchicine because of their significantly increased mitotic rate (32). Albendazole and mebendazole are anthelmintics used to treat worm infections. According to our research, albendazole and mebendazole displayed cytotoxic effects similar to those of colchicine. Similarly, albendazole and mebendazole act by binding to the colchicine-sensitive site on β-tubulin, thus blocking its assembly into microtubules (33, 34). These findings strongly support that the proposed underlying mechanism has inhibitory effects on tubulin polymerization and provide convincing evidence for the anticancer effects of colchicine in osteosarcoma. Owing to a higher bioavailability, long-term half-life and several advanced research reporting anticancer effects of benzimidazoles in osteosarcoma cell lines, albendazole, and mebendazole were also excluded (11, 33, 35–38).

Accumulating evidence suggests that cancer cells acquire resistance mechanism to chemotherapy over time during treatment, eventually leading to cancer recurrence and relapse (39). This phenomenon accounts for a major problem in the treatment of cancer. In osteosarcoma, about 15%–20% of patients display evidence of metastases at initial diagnosis, mostly in the lungs (40). Therefore, complete surgical resection of all clinically detected tumor sites is mandatory, and chemotherapy is required for most patients. However, the overall survival rate remains stagnant. Therefore, it has been suggested that the therapeutic effects of traditional surgical and chemo/radiotherapy could be improved by employing new or combinational treatments targeting osteosarcoma. Combinations of gemcitabine and cisplatin with colchicine derivative have demonstrated synergism in urothelial carcinoma (41). In addition, colchicine induces autophagy and senescence in lung cancer cells, suggesting that it may have potential for use in combination with autophagy inhibitors for cancer therapy (42). In this context, colchicine is a promising candidate for use in combination with synthetic chemotherapeutic drugs.

Most cancers involve p53 mutations and genomic instability. p53 is a critical tumor suppressor gene that modulates checkpoint responses to DNA damage. p53 mutations are reported to be significantly associated with considerable levels of genomic instability in osteosarcoma (43, 44). A meta-analysis of small-sized studies suggested that p53 mutations in osteosarcoma demonstrated an unfavorable outcome on 2-year overall survival in comparison with that of the wild-type p53 (45). According to previous reports, p53 is a powerful prognostic indicator of osteosarcoma. In addition, Tang et al. suggested that mutant p53 is a promising target for osteosarcoma (46). To validate the correlation between wild-type and mutant osteosarcoma, we conducted experiments using Saos-2 p53^-/-^ cells and U2OS p53^+/+^ cells throughout the study. These results indicated that colchicine exerted cytotoxicity in both p53-deficient and wild-type osteosarcoma cells. Given that preceding research suggests that the p53 mutation is a key indicator and therapeutic target of osteosarcoma, the results from our study demonstrate the anticancer effects of colchicine, implying its clinical importance in osteosarcoma patients, regardless of p53 mutation. However, whether colchicine is superior to the current chemotherapeutics used to treat osteosarcoma needs to be explored. The drugs used most often to treat osteosarcoma include adriamycin, cisplatin, methotrexate, ifosfamide, and epirubicin (47). Usually, two or more drugs are given together before surgery in very high doses. In this manner, the anticancer and side effects of colchicine compared to those of other drugs should be studied. In addition, the underlying mechanism by which colchicine regulates the cell cycle of osteosarcoma cell lines requires a further study. We have demonstrated that colchicine induces apoptosis and inhibits the cell cycle of osteosarcoma. The signaling pathways modulated by colchicine and inter-cell line variation between Saos-2 and U2OS cells with detailed mechanisms remain a task.

## Conclusion

Our findings suggest that colchicine is a potential agent that exerts cytotoxic effects against osteosarcoma. The anticancer effects of colchicine outside the scope of its original medical indication suggest that colchicine could be developed as a cost-effective and timesaving chemotherapeutic agent. The mechanism by which colchicine modulates the signal transduction pathway involved in microtubule polymerization requires further investigation.

## Data availability statement

The original contributions presented in the study are included in the article/**Supplementary Material**. Further inquiries can be directed to the corresponding author.

## Ethics statement

The animal study was reviewed and approved by CHA IACUC.

## Author contributions

JO: Project administration, data curation, formal analysis, and writing original draft. HA: Data curation, formal analysis, and writing original draft. HY: Data curation and formal analysis. SC: Data curation and formal analysis. JSO: Manuscript review and editing. SK: Data curation. JK: Data curation. JC: Data curation. SL: Conceptualization, funding acquisition, investigation, methodology, manuscript review, and editing. All authors contributed to the article and approved the submitted version.

## Funding

This work was supported by the Korea Health Technology R&D Project through the Korea Health Industry Development Institute. It was funded by the Ministry of Health and Welfare, Republic of Korea (grant number HI16C1559), and by the National Research Foundation of Korea (NRF) grant funded by the Korean government (MSIT) (Nos. NRF-2021R1A4A3023587 and 2022R1A2C2005916).

## Conflict of interest

The authors declare that the research was conducted in the absence of any commercial or financial relationships that could be construed as a potential conflict of interest.

## Publisher’s note

All claims expressed in this article are solely those of the authors and do not necessarily represent those of their affiliated organizations, or those of the publisher, the editors and the reviewers. Any product that may be evaluated in this article, or claim that may be made by its manufacturer, is not guaranteed or endorsed by the publisher.

## Supplementary material

Supplementary Figure 1 Initial screening of 772 FDA-approved library compounds in graphical visualization. The cell viability was assessed by CCK-8 assays in **(A)** Saos-2 and **(B)** U2OS cells with a concentration of 10 μM for 48 h. Arrows represent the most effective 30 non-chemotherapeutics in each cell line.

Supplementary Table 1 Initial screening of 772 FDA-approved library compounds. The table represents the number of FDA-approved drugs, name of the compounds and the viability of Saos-2 and U2OS cells with colchicine treatment of 10 μM for 48 h.

Supplementary Table 2 A list of the deferentially expressed genes between colchicine treated and non-treated Saos-2 and U2OS cells.
