# Supplementary material for: Colchicine as a novel drug for the treatment of osteosarcoma through drug repositioning based on an FDA drug library
Source: Front Oncol. 2022 Aug 18;12:893951. doi: 10.3389/fonc.2022.893951 (PMC9433722; doi:10.3389/fonc.2022.893951)

**Supplementary materials.**

**Colchicine as a novel drug for the treatment of osteosarcoma through drug repositioning based on an FDA drug library**

**Jisun Oh^1^, Hyun‑Ju An^1^, Hyun Jeong Yeo^1^, Sujin Choi^1^, Jisu Oh^2^, Segi Kim^1^, Jin Man Kim^3^, Junwon Choi^4^, Soonchul Lee^1*^**

^1^Department of Orthopaedic Surgery, CHA Bundang Medical Center, CHA University School of Medicine, 335 Pangyo-ro, Bundang-gu, Gyeonggi-do, Seongnam-si, 13488, Republic of Korea.

^2^Division of Hemato-Oncology, Department of Internal Medicine, Yongin Severance Hospital, Yonsei University College of Medicine, 363 Dongbaekjukjeon-daero, Giheung-gu, Yongin-si 16995, Korea.

^3^Department of Oral Microbiology and Immunology School of Dentistry, Seoul National University, **1 Gwanak-ro, Gwanak-gu, Seoul, 08826, Republic of Korea**

**^4^Department of Molecular Science and Technology, Ajou University, Suwon-si, Gyeonggi-do 16499, Republic of Korea**

***Correspondence**:

Professor Soonchul Lee

Department of Orthopaedic Surgery, CHA Bundang Medical Center, CHA University School of medicine, Pangyo-ro, Bundang-gu, Geyonggi-do, seongnam-si, 13488, Republic of Korea

Tel.:+82-31-780-5289; Fax: +82-31-708-3578

E-mail: lsceline78@gmail.com

**Supplementary Fig. S1.** Initial screening of 772 FDA-approved library compounds. The cell viability was assessed by CCK-8 assays in (A) Saos-2 and (B) U2OS cells with a concentration of 10 μM for 48 h. Arrows represent the most effective 30 non-chemotherapeutics in each cell line.

**(A)**


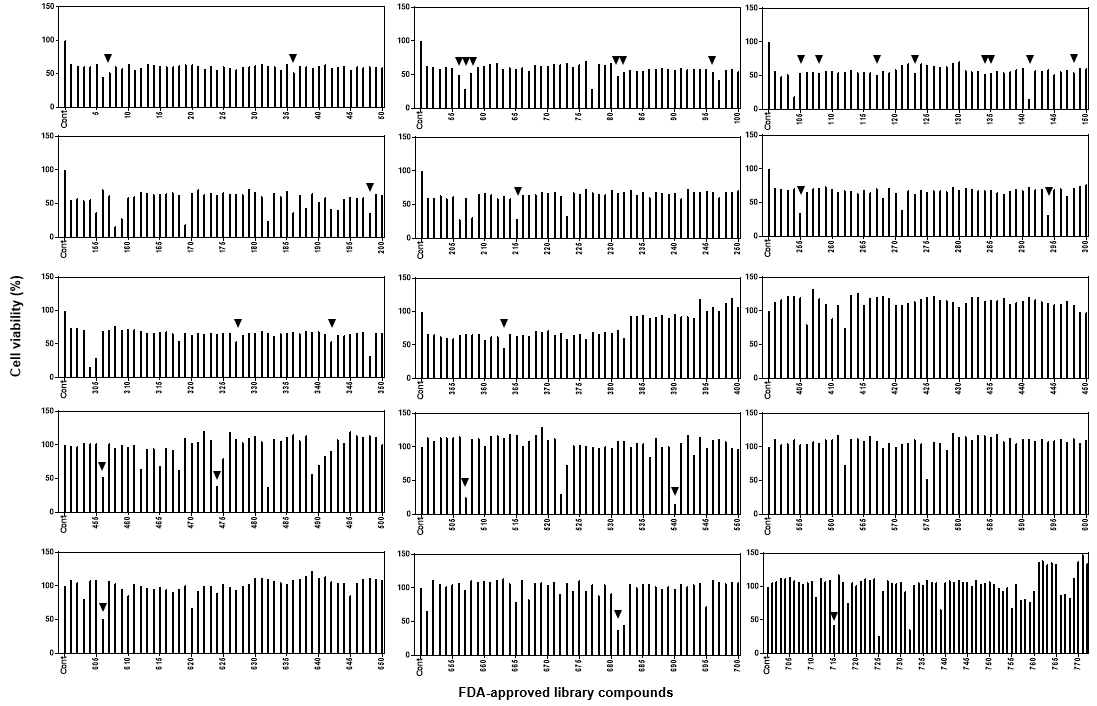


**(B)**


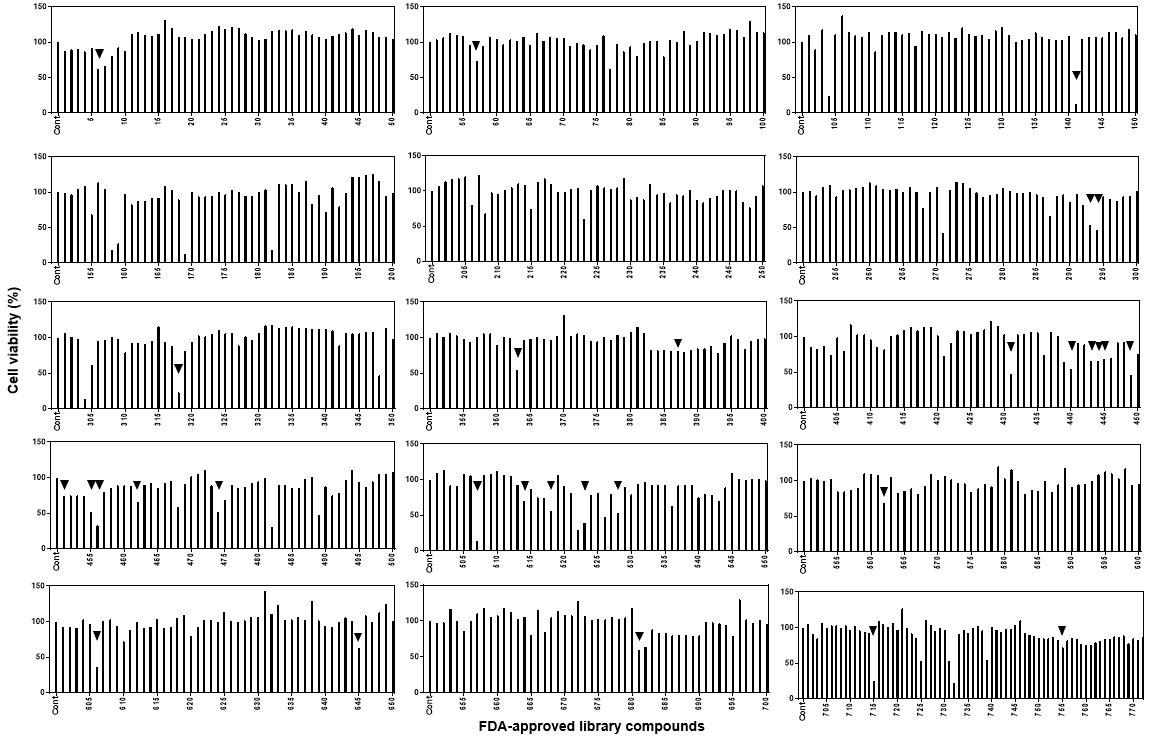

Supplement: Supplementary Figure 1 — Initial screening of 772 FDA-approved library compounds in graphical visualization. The cell viability was assessed by CCK-8 assays in (A) Saos-2 and (B) U2OS cells with a concentration of 10 μM for 48 h. Arrows represent the most effective 30 non-chemotherapeutics in each cell line. [file DataSheet_1.docx]
